# Knowledge, Attitudes, and Practices Toward COVID-19 Among the General Public in the Border Region of Jazan, Saudi Arabia: A Cross-Sectional Study

**DOI:** 10.3389/fpubh.2021.733125

**Published:** 2021-12-08

**Authors:** Mohammed J. Almalki

**Affiliations:** Assistant Professor of Health Services Management and Policy, College of Public Health and Tropical Medicine, Jazan University, Jazan, Saudi Arabia

**Keywords:** knowledge, attitudes, practices, COVID-19, novel coronavirus disease, SARS-CoV-2, Jazan, Saudi Arabia

## Abstract

**Background:** The Saudi government had implemented unprecedented preventive measures to deal with COVID-19. These measures included intermittent curfews, bans on public gatherings, limitations on many services, temporary suspension of Hajj, Umrah, and visit and launching awareness campaigns. Therefore, this study aimed to assess the KAP toward COVID-19 among residents of the border region of Jazan, Saudi Arabia.

**Methods:** An online cross-sectional survey was distributed from May 4 to May 21, 2020, using a Google Form. The survey questionnaire covered demographic characteristics and KAP toward COVID-19. The KAP questions consisted of 17 items on knowledge, four items on attitude, and six items on practice.

**Results:** A total of 597 participants responded to the survey questionnaire. Overall, participants demonstrated a good knowledge of COVID-19, correctly answering 77% of the knowledge questions. Most of the participants exhibited good attitudes and acceptable practices toward COVID-19. Multiple regression analysis revealed that participants with a university education (B = 1.75) or post-graduate education (B = 2.24), those with an income >SR 10,000–20,000 (B = 1.38) or >SR 20,000 (B = 2.07), and those who had received a personal health education (B = 1.19) had higher COVID-19 knowledge scores (*p* < 0.05). The ordinal logistic regression analysis found that compared to being female, being male was significantly associated with worrying about COVID-19 (*p* = 0.024, OR = 1.78), willingness to receive a COVID-19 vaccine (*p* = 0.003, OR = 1.81), and willingness to report potential symptoms of COVID-19 (*p* = 0.046, OR = 2.28). Worrying about COVID-19 was significantly associated with pre-university education vs. post-graduate education (*p* ≤ 0.001, OR = 7.94) and university education vs. post-graduate education (*p* ≤ 0.001, OR = 4.17). The binary logistic regression analysis found that compared to being female, being male was significantly associated with less face mask wearing in public (*p* = 0.009, OR = 0.31): Females were 3.23 times more likely to wear a face mask than were males.

**Conclusions:** Most of the study participants had good knowledge, positive attitudes, and effective practices toward COVID-19. The findings of this study may help guide future awareness resources to the groups most in need in the Jazan region, particularly as the COVID-19 situation develops and changes. Further assessment should consider the groups omitted from this study, including immigrants and the elderly who have not adopted social media and technology.

## Introduction

In 2020, the World Health Organization (WHO) announced COVID-19 as a novel respiratory disease caused by the coronavirus SARS-CoV-2 that emerged in Wuhan, China (1, 2). COVID-19 spread quickly around the world, resulting in many infections, and deaths. As a result, hospitals and health services providers have faced critical pressure in meeting people's healthcare needs. As of June 25, 2021, there had been a total of 179,686,071 confirmed cases of COVID-19 worldwide, with 3,899,172 confirmed deaths (3).

In response to the wide spread of COVID-19, the WHO declared it a global pandemic in March 2020 and called for global collaboration (1, 2). Following the WHO declaration and the confirmation of the first COVID-19 case in Saudi Arabia on March 2, 2020, the Saudi government took strict and unprecedented prevention measures against COVID-19 (4–7). These measures were aimed to control the disease spread and to maintain people's health and safety. These preventive measures included a travel ban to and from Saudi Arabia; intermittent curfews; bans on public gatherings; and the closure of most government departments, universities, schools, malls, and mosques. Hajj, Umrah, and visit visas were also temporarily suspended. In addition, national health awareness campaigns were launched using various means of communication. Despite considerable government efforts to control COVID-19, the number of confirmed cases had reached 479,390, with 7,730 deaths, as of May 28, 2021 (3). Recently, Saudi Arabia reported 16,893 new COVID-19 cases over 14 days (June 10–23), the highest in many months. As of June 26, 2021, there were 11,331 active cases in Saudi Arabia, and 7% of these were from the Jazan region (8). In addition, 436 violations of COVID-19 precautionary measures were recorded among Jazan residents from June 6 to June 12, 2021 (9).

Adherence to the precautionary measures is essential to control the pandemic; however, adherence is influenced by people's knowledge, attitudes, and practices (KAP) toward COVID-19 (10, 11). A number of studies on COVID-19 KAP were conducted worldwide. For example, a Chinese survey study conducted among 6,910 participants showed that the correct overall rate of the knowledge questionnaire was 90%. In addition, 97.1% of the participants had confidence in the government COVID-19 competing efforts, and 98.0% committed to preventive measures such as wearing masks when going out (10). Another cross-sectional study was conducted to assess the KAP toward COVID-19 among 3,712 Egyptians. Participants reported sufficient knowledge (75.9%), a positive attitude (70.2%), and good practice (49.2%) (12).

Many COVID-19 KAP studies were also conducted in the general public of Saudi Arabia. A recent study found that most participants demonstrated good KAP levels: 89.6% for knowledge, 87.2% for attitudes, and 87.2% for practices. However, most of the participants were recruited from the central, eastern, and western regions, with only 86 out of 1,513 participants from the whole southern region, which includes Jazan (13). Another cross-sectional study found that levels of KAP toward COVID-19 were 81.3% for knowledge, 86.6% for attitudes, and 81.9% for practices (14). This study, too, had few participants from the Jazan region: only 36 out of 1,513 participants. The majority of their participants were from the Aseer, Makkah, Eastern, and Riyadh regions. In addition, Al-Hanawi et al., conducted a survey study to investigate the KAP of the Saudi public toward COVID-19, revealing that the majority of participants exhibited a high level of knowledge (28.23, SD = 2.76, range: 6–30), held optimistic attitudes (17.96, SD = 2.24, range: 3–22), and engaged in good preventive practices (4.34, SD = 0.87, range: 0–5). However, there were only 19 participants from the Jazan region compared with 2,257 individuals from the Western region (11). Another study from Jeddah, Saudi Arabia, found that 68.1% of the participants had good knowledge scores, 93.1% had a positive attitude, and 97.7% demonstrated good preventive practices against COVID-19 (15). Overall, previous COVID-19 KAP studies from Saudi Arabia presented limited or no data on the border region of Jazan.

The Jazan region is located in southern Saudi Arabia and has the highest population density among other regions. Jazan region has had a substantial development in all aspects in the last decade. However, it cannot be compared to other regions such as Riyadh, Makkah, Madinah, Qassim, Eastern, and Asir. Moreover, close to the border with Yemen, its location renders it vulnerable to illegal migration from Yemen and the countries in the Horn of Africa (16). Such movements also make Jazan more vulnerable to cross-border transmitted infectious diseases, including COVID-19. In 2000, the Jazan region suffered from human and livestock infection caused by Rift Valley Fever. This was the first time this arbovirus had been identified outside Africa and Madagascar (17). Similarly, in 2017, cholera was detected in a group of illegal immigrants to Jazan, making the local population vulnerable to this infectious disease (18).

Assessing KAP toward COVID-19 in the general public of Jazan is essential for understanding the current situation and identifying gaps for system and policy improvement, including the rational utilization of educational health resources. In addition, KAP studies from Jazan will add to the current body of knowledge and serve as a baseline for future COVID-19 research projects in the region. Therefore, this study aims to assess the KAP toward COVID-19 among the general public in the border region of Jazan, Saudi Arabia.

## Materials and Methods

### Design and Sample

This study used a cross-sectional design using a non-probability sampling technique. The required sample size was determined to be 384 using Epi Info version 7.2.4 (19). The equation to determine sample size included the population of Jazan (1,365,110) (20), a 50% response distribution, a 5% margin of error, and a 95% confidence interval.

### Questionnaire

The author designed a KAP questionnaire toward COVID-19 to fit the local context using several scientific resources on COVID-19 published by the Ministry of Health in Saudi Arabia, the Public Health Authority, the WHO, the United States Centers for Disease Control and Prevention (CDC), and the study by Zhong et al., (10, 21–24). The questionnaire included two sections: demographics and COVID-19 KAP. The demographic variables consisted of age, gender, marital status, education level, occupation, income per month, area of residency, nationality, sources of information, and whether the participant had received a personal health education (face-to-face or online health education activities). The KAP section included 27 items. The knowledge dimension included 17 items. The answer options for the knowledge scale items K.1–K.7 were yes, no, and not sure, with the K.2 and K.3 scale items being reverse scored. The knowledge scale items K.9–K.17 were answered by selecting the appropriate answer/answers from a list of possibilities. A correct answer was given one point, and an incorrect answer or a response of not sure was assigned 0. The range of the knowledge scores for each participant was 0–38—the higher the knowledge score, the higher the knowledge of COVID-19. The attitudes dimension consisted of four questions on a five-point Likert scale. The responses included strongly agree, agree, not sure, disagree, and strongly disagree, which were coded from 5 to 1, respectively. The practices dimension consisted of six yes/no questions coded one for yes, 0 for no.

Two public health experts assessed and validated the instrument, providing several suggested modifications to improve the content and clarity. The author translated the questionnaire to Arabic, and two bilingual researchers ensured its clarity. The questionnaire was tested on a pilot sample to ensure its accuracy, after which two questions were modified to simplify their language. Cronbach's alpha for the knowledge scale in this study was 73, which is acceptable (25).

### Data Collection

An online survey was distributed from May 4 to May 21, 2020, using a Google Form that took approximately 6 min to complete. The author distributed the survey link to his contacts on WhatsApp and followers on Twitter. The participants were asked to pass the survey link to their contacts and followers on social media platforms. The eligibility criteria for participation were being a resident of the Jazan region aged 18 years or older who agreed to participate in the survey. Each participant was permitted to submit one questionnaire per IP address to avoid duplicate submissions. Informed consent was obtained from all participants through a yes/no question on the first page of the questionnaire. Participants were also informed about the voluntary nature of their participation, the steps taken to maintain the confidentiality of the data, and how to complete the online survey. The Research Ethics Committee of Jazan University, Saudi Arabia, approved this study (REC41/5/094).

### Data Analysis

The data were entered, cleaned, coded, and analyzed using IBM SPSS Statistics software version 27 for Windows. Frequencies and descriptive statistics of COVID-19 KAP were calculated and reported. Multiple regression was applied to identify possible independent demographic variables associated with participants' knowledge scores as a dependent variable. In addition, binary and ordinal logistic regressions were utilized to check associations of the independent demographic variables with the dependent variables of attitudes and practices as appropriate. The independent variables for all models included gender, age, education level, occupation, income per month, whether the participant had received a personal health education, and area of residency (rural or urban). These independent variables were entered as a set into all models of the regression analyses. For the regression analyses, the independent variables were regrouped from 5 to 3 groups: (Strongly Agree and Agree to Agree = 3), (Neutral = 2), and (Strongly Disagree and Disagree to Disagree = 1). Statistical tests with *p* ≤ 0.05 were considered statistically significant.

## Results

**Figure 1:**
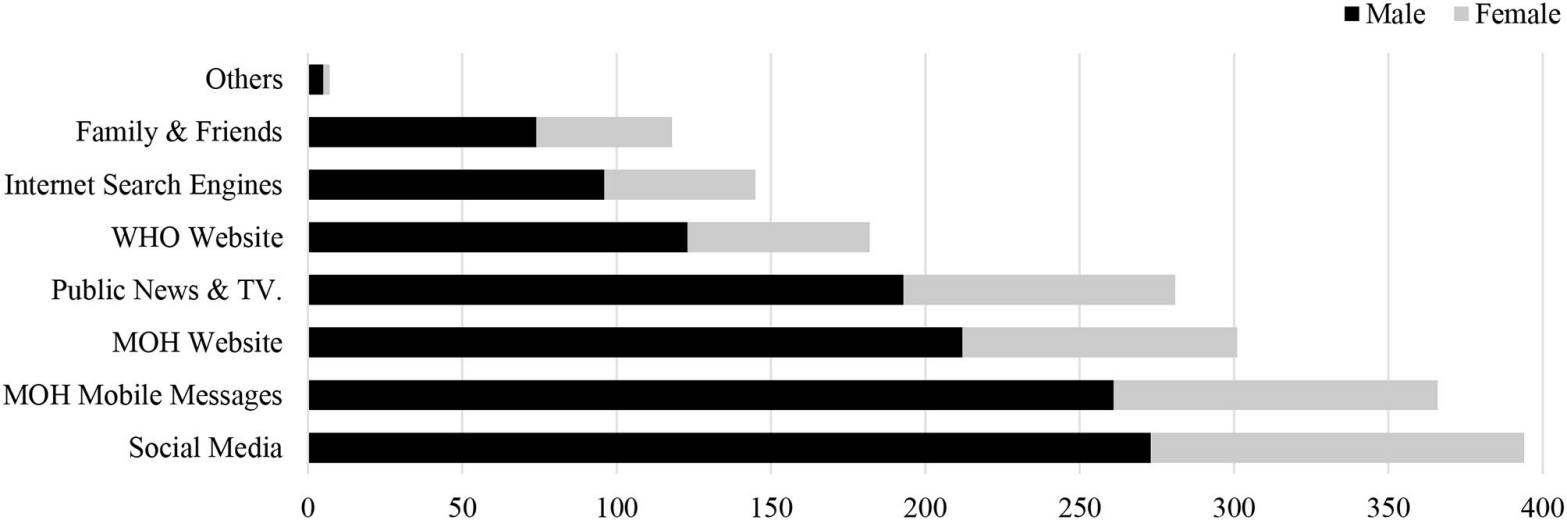
COVID-19 information sources by gender.

A total of 597 participants responded to the survey, with an average age of 35.35 years (SD = 9.98, range 18–60). Most of participants were male (414; 69.3%), Saudi (534; 89.4%), married (408; 68.3%), from urban areas (350; 58.6%), held a university degree (415; 69.5%), employed in a non-health job (304; 50.9%, Table 1), and received most of the COVID-19 information from social media (394; 66.0%) and Ministry of Health mobile messages (366; 61.3%; Figure 1).

**Table 1:** Demographic characteristics of participants.

**Variables**		***N***	**%**
**Gender**	Male	414	69.3
	Female	183	30.7
**Age**	<30 years	187	31.3
	30–40 years	240	40.2
	>40 years	170	28.5
**Marital status**	Married	408	68.3
	Not married	189	30.3
**Nationality**	Saudi	534	89.4
	Non-Saudi	63	10.6
**Education level**	Pre-university	98	16.4
	University	415	69.5
	Post-graduate	84	14.1
**Occupation**	Health related	79	13.2
	Non-health related	304	50.9
	Unemployed	214	35.8
**Income per month**	≤ SR 10,000	291	48.7
	>SR 10,000–20,000	264	44.2
	>SR 20,000	42	7.0
**Health education**	No	328	54.9
	Yes	269	45.1
**Area of residency**	Rural	247	41.4
	Urban	350	58.6

### Knowledge

The mean COVID-19 knowledge score was 29.74 out of 38 (SD = 4.03, range 18–38), indicating an overall knowledge level of 78% (Table 2).

**Table 2:** Responses to questions about COVID-19 knowledge.

***N* = 597**		**Correct answer**	**(%)**	
K.1		COVID-19 spread worldwide.	594	(99.5)
K.2		Availability of COVID-19 vaccine.	458	(76.7)
K.3		All cases of COVID-19 become severe.	408	(68.3)
K.4		Risk of contracting COVID-19 increases in mass gatherings.	583	(97.7)
K.5		Confirmed COVID-19 cases have occurred in Saudi Arabia.	578	(96.8)
K.6		It is believed that COVID-19 emerged from an animal source.	451	(75.5)
K.7		Availability of COVID-19 diagnostic tests.	547	(90.6)
K.8		COVID-19 appeared for the first time in: (Wuhan, China).	572	(95.8)
K.9		The cause of COVID-19 is: (Virus).	528	(88.4)
K.10		The confirmed incubation period of COVID-19 is: (2-14 days).	574	(96.1)
**K.11 COVID-19 symptoms may include:**				
	K.11a)	Fever.	556	(93.1)
	K.11b)	Dry cough.	543	(91.0)
	K.11c)	Shortness of breath.	577	(96.6)
	K.11d)	Tiredness.	328	(54.9)
	K.11e)	Sore throat.	380	(63.7)
	K.11f)	Congested or runny nose.	217	(36.3)
	K.11g)	Diarrhea.	182	(30.5)
	K.11h)	Loss of taste or smell.	217	(33.3)
**K.12 Groups at increased risk of COVID-19 are:**				
	K.12a)	Older people.	560	(93.8)
	K.12b)	People with pre-existing medical conditions.	533	(89.3)
	K.12c)	Pregnant women.	237	(39.7)
**K.13 Availability of specific medication for COVID-19:**				
	K.13a)	There is no specific medication.	371	(62.1)
	K.13b)	Only health care with supportive medications.	383	(64.2)
**K.14 In severe cases of COVID-19, infection can cause:**				
	K.14a)	Pneumonia.	231	(38.7)
	K.14b)	Acute respiratory distress syndrome.	249	(41.7)
	K.14c)	Death.	496	(83.1)
**K.15 People should wear face masks:**				
	K.15a)	If they have respiratory symptoms (coughing/sneezing).	546	(91.5)
	K.15b)	When in contact with people with respiratory symptoms.	561	(94.0)
**K.16 Anyone with possible COVID-19 symptoms must:**				
	K.16a)	Wear a face mask.	403	(67.5)
	K.16b)	Isolate himself/herself away from others.	562	(94.1)
	K.16c)	Call the Ministry of Health hotline (937).	562	(94.1)
	K.16d)	Visit the nearest health facility, taking precautions meanwhile.	420	(70.4)
**K.17 Prevention measures against COVID-19 may include:**				
	K.17a)	Wash or sanitize hands as recommended.	583	(97.7)
	K.17b)	Avoid touching the face with unwashed hands.	576	(96.5)
	K.17c)	Avoid contact with infected people.	569	(95.3)
	K.17d)	Cover mouth and nose when coughing or sneezing.	551	(92.3)
	K.17e)	Avoid shaking hands.	569	(95.3)
	K.17f)	Wash or sanitize hands after touching objects in public.	501	(83.9)

*N = total number of participants; n = number of participants who answered correctly; and % = percentage of those with the “correct answer” compared to the total sample*.

### Factors Associated With COVID-19 Knowledge

Multiple regression analysis showed that participants with a university education (B = 1.75) or post-graduate education (B = 2.24), those with a monthly income >SR 10,000–20,000 (B = 1.38) or >SR 20,000 (B = 2.07), and those who received a personal health education (B = 1.19) had higher COVID-19 knowledge scores (*p* < 0.05). The overall regression model was statistically significant (*F*_(11,585)_ = 7.72, *p* < 0.001, *R*^2^ = 0.127), and the predictors as a set explained 12.7% of the variance in knowledge scores (Table 3).

**Table 3:** Standard multiple regression analysis of COVID-19 knowledge and demographic predictors.

							**95% CI for B**	
		**B**	**SE B**	**Beta**	**t**	**Sig**.	**Lower**	**Upper**
**(Constant)**		26.793	0.519		51.662	<0.001^**^	25.775	27.812
**Gender**	1 = Female	−0.568	0.367	−0.065	−1.548	0.122	−1.289	0.153
**Age**	1 = <30 yrs.	−0.215	0.474	−0.025	−0.453	0.650	−1.147	0.717
	3 = >40 yrs.	−0.236	0.406	−0.026	−0.582	0.561	−1.033	0.561
**Education level**	2 = University	1.747	0.464	0.200	3.761	<0.001^**^	0.835	2.659
	3 = Post-graduate	2.244	0.664	0.194	3.377	0.001^**^	0.939	3.549
**Occupation**.	1 = Health related	0.742	0.516	0.062	1.438	0.151	−0.272	1.755
	3 = Unemployed	0.508	0.467	0.061	1.089	0.277	−0.408	1.425
**Income per month**	2 = >SR 10,000–20,000	1.381	0.432	0.170	3.199	0.001^**^	0.533	2.228
	3 = >SR 20,000	2.071	0.740	0.132	2.799	0.005^*^	0.617	3.524
**Health education**	1 = Yes	1.194	0.339	0.148	3.521	<0.001^**^	0.528	1.860
**Area of residency**	1 = Urban	0.261	0.352	0.032	0.741	0.459	−0.430	0.951

*Method: Enter. Dependent variable: total scores of knowledge*.

* *p < .05*.

** *p ≤ .001. R^2^ =.127 (Adjusted R^2^ =.110). References groups were excluded automatically by SPSS: Gender (0 = Male), Age (2 = 30–40 yrs.), Education level (1 = Pre-university), Occupation (2 = Non-health related), Income per month (1 =≤SR 10,000), Health education (0 = No), Area of residency (0 = Rural)*.

### Attitudes and Practices

Regarding attitudes, most participants (97.0%) were worried about contracting COVID-19, and 84.6% expressed willingness to get the potential COVID-19 vaccine (at the time of the survey, vaccines had not yet been developed). Almost all participants (99.2%) expressed willingness to report COVID-19 symptoms to the health authorities, and 91.0% were confident in government efforts against COVID-19 (Table 4).

**Table 4:** Attitudes toward COVID-19.

	**Items (N = 597)**	**Agree**		**Not Sure**		**Disagree**	
		***n***	**(%)**	***n***	**(%)**	***n***	**(%)**
**Attitude 1**	Worrying about contracting COVID-19.	579	(97.0)	14	(2.3)	4	(0.7)
**Attitude 2**	Willingness to receive COVID-19 vaccine.	505	(84.6)	75	(12.6)	17	(2.8)
**Attitude 3**	Willingness to report COVID-19 symptoms.	592	(99.2)	5	(0.8)	0	(0.0)
**Attitude 4**	Confidence in government efforts during COVID-19.	543	(91.0)	45	(7.5)	9	(1.5)

*N, total number of participants; answer options, Agree (Agree & Agree Strongly), Not Sure, and Disagree (Disagree and Strongly Disagree); n, number of responses to each item; %, percentage of the responses to each item*.

Regarding practices related to COVID-19, 98.8% of the participants claimed that they were maintaining social distancing, 98.3% avoided leaving the house, and 96.0% were washing/sanitizing their hands according to protocol. In addition, the majority of participants (95.5%) indicated that they avoided touching the face with unwashed hands, 95.8% avoided shaking hands, and 91.6% wore face masks in public (Table 5).

**Table 5:** Practices related to COVID-19.

	**Items (*N* = 597)**	**Yes**		**No**	
		***n***	**(%)**	***n***	**(%)**
**Practice 1**	Avoiding leaving the house except for necessities.	587	(98.3)	10	(1.7)
**Practice 2**	Wearing a face mask in crowded places.	547	(91.6)	50	(8.4)
**Practice 3**	Avoiding shaking hands with others.	572	(95.8)	25	(4.2)
**Practice 4**	Maintaining social distancing.	590	(98.8)	7	(1.2)
**Practice 5**	Avoiding touching the face with unwashed hands.	570	(95.5)	27	(4.5)
**Practice 6**	Washing or sanitizing hands as recommended.	573	(96.0)	24	(4.0)

*N, total number of participants; answer options, yes, no; n, number of responses to each item; %, percentage of the responses to each item*.

### Factors Influencing COVID-19 Attitudes and Practices

Ordinal logistic regression analysis found that being males was significantly associated with worrying about COVID-19 (*p* = 0.024, OR = 1.78), willingness to take a vaccine (*p* = 0.003, OR = 1.81), and willingness to report symptoms (*p* = 0.046, OR = 2.28). Furthermore, compared to post-graduate education, worrying about COVID-19 was significantly associated with pre-university education (*p* ≤ 0.001, OR = 7.94), and university education (*p* ≤ 0.001, OR = 4.17).

Binary logistic regression analysis found that being male was significantly associated with less face mask wearing in public (*p* = 0.009, OR = 0.31): Women were 3.23 times more likely to wear a face mask than were men (Table 6).

**Table 6:** Ordinal and binary logistic regression analysis on factors significantly associated with attitudes and practices toward COVID-19.

**Variable**		**B**	**Std. Error**	**Wald**	**df**	**Sig**.	**Odds ratio**	**95% CI**	
								**Lower**	**Upper**
**Attitude 1: Worrying about COVID-19**									
Gender	0 = Male	0.576	0.256	5.072	1	0.024^*^	1.78	0.075	1.078
	1 = Female	0a	0.0	0.0	0	0.0		0.0	0.0
Edu. level	1 = Pre-university	2.072	.502	17.001	1	<0.001^**^	7.94	1.087	3.056
	2 = University	1.429	0.317	20.371	1	<0.001^**^	4.17	0.809	2.050
	3 = Post-graduate	0a	0.0	0.0	0	0.0		0.0	0.0
**Attitude 2: Willingness to receive COVID-19 vaccine**									
Gender	0 = Male	0.596	0.198	9.083	1	0.003^*^	1.81	0.208	0.984
	1 = Female	0a	0.0	0.0	0	0.0		0.0	0.0
**Attitude 3: Willingness to report COVID-19 symptoms**									
Gender	0 = Male	0.822	0.413	3.968	1	0.046^*^	2.28	0.013	1.631
	1 = Female	0a	0.0	0.0	0	0.0		0.0	0.0
**Practice 2: Wearing a face mask in crowded places**									
Gender	0 = Male	−1.160	0.443	6.869	1	0.009^*^	0.31	0.132	0.746
	1 = Female	0a	0.0	0.0	0	0.0		0.0	0.0

*Method: Enter*.

*a, This parameter is set to zero because it is redundant; In OLR dependent variables are (Attitude 1): worrying about COVID-19, (Attitude 2): willingness to take COVID-19 vaccine, and (Attitude 3): willingness to report COVID-19 symptoms; In BLR dependent variable is (Practice 2), wearing a face mask in crowded places; Only significant coefficients are presented in this table*.

* *p < 0.05*.

** *p < 0.001*.

## Discussion

Countries worldwide have made many efforts to confront COVID-19, yet it continues to pose a threat to all aspects of people's lives. Understanding and improving people's KAP regarding COVID-19 are essential to effectively prevent and control COVID-19 (26). The current study aimed to assess the COVID-19 KAP among residents of the Jazan region in Saudi Arabia.

Knowledge of COVID-19 was relatively good in the Jazan community. This result was, to some extent, consistent with previous findings from Saudi Arabia (14) but higher than findings from Ethiopia (26) and China (27). It was expected that the knowledge of Jazan population would be less than that of the main regions of Saudi Arabia. This is because Jazan is a border region with many remote areas and illegal immigrants. Interestingly, no differences were revealed in KAP toward COVID-19 according to the area of residency (rural or urban). This finding is different from that of prior studies in which rural residents were more likely to have poor KAP toward COVID-19 than urban residents (10, 26). This may be due to ongoing awareness campaigns by the health authorities through social media, mass media, and mobile messages. Another possible reason is that the participants in this study had Internet access, making awareness information accessible regardless of where they lived. Another possible explanation is that the sample of this study consisted of educated participants only.

In line with previous literature (13, 27, 28), social media platforms were a source of information regarding COVID-19 for the majority of the participants. Although social media platforms provide easily accessible information, they can also be a source of misinformation (29). A cross-sectional survey from Jeddah, Saudi Arabia, found that various misconceptions regarding COVID-19 were present among two-thirds of their study participants. Their findings also revealed that social media was the leading source of information for their participants (15).

The majority of the participants were worried about contracting COVID-19 and willing to receive the COVID-19 vaccine. While this finding is consistent with several prior studies (27, 30), it is inconsistent with others (31, 32). It can be argued that worrying about COVID-19 may reinforce one's sense of personal and societal responsibility to adhere to appropriate preventive practices and thus contribute to combating the pandemic. The willingness of the participants to receive the COVID-19 vaccine is encouraging, especially in light of the COVID-19 vaccine hesitancy worldwide (33–37). Reasons for such hesitancy were rumors, conspiracy theories, and false claims about COVID-19 vaccines, including their purpose, contents, efficacy, safety, and side effects (38, 39). Previous studies from Saudi Arabia found that most of their participants were hesitant to receive a COVID-19 vaccine (40, 41). However, a recent study from the Jazan region indicated that the COVID-19 vaccination had a high level of acceptance (42). In addition, recent statistics showed that nearly 16 million doses of COVID-19 vaccines had been administered in Saudi Arabia by mid-June 2021 (43). These figures are encouraging, as they indicate the success of precautionary efforts undertaken by the government and health authorities to enhance vaccine acceptability among the population. Another explanation is the high confidence in the government's efforts during COVID-19 (11). These efforts include the well-equipped screening centers, free treatment for Saudis and non-Saudis, and COVID-19 vaccination for all residents, including illegal immigrants (44).

The study participants reported good compliance with social distancing, washing and sanitizing hands, avoiding leaving the house except for necessities, avoiding shaking hands with others, avoiding touching their faces with unwashed hands, and wearing face masks in public. Most families in Saudi Arabia are multigenerational families, and they have close social ties; thus, they often exchange visits and meetings. Hand-shaking is also a cultural behavior of significant value among Saudis (45). The fact that most participants reported distancing themselves from these behaviors indicates a positive attitude and practice that may assist in combating COVID-19. The rates reported in the current study were higher than those of a previous Saudi survey, which found that its participants demonstrated some knowledge gaps and negligence in hand hygiene practice (46). The majority of the current study stated that they wore face masks in public, although this was not required during the data collection period. At the time, the WHO and the Ministry of Health encouraged people to avoid wearing face masks, except those who had symptoms of COVID-19 or those caring for infected people (47, 48). At present, using face masks in Saudi Arabia in public is compulsory, and those who do not adhere to this face charges (49).

The study findings revealed some predictors associated significantly with the COVID-19 KAP of the participants. Similar to previous studies (10, 11, 50), participants with a middle or high income, and those who had received a personal health education indicated a higher level of COVID-19 knowledge than others. Moreover, the higher the education level, the higher the COVID-19 knowledge (15, 27, 29). On the other hand, the knowledge of health-related personnel was not significantly different from that of others, inconsistent with previous studies (10, 27). Possible causes for this difference are the effective COVID-19 awareness campaigns and the availability of the Internet and smart devices for the participants in our study, making it easier for them to acquire information about COVID-19.

Compared to female participants, male participants showed more concern about COVID-19, a higher willingness to receive the vaccine, and a higher willingness to report possible symptoms of COVID-19 to health authorities. These findings diverge from those of prior studies (11, 15), in which females showed better attitudes toward COVID-19 than men. Perhaps female participants in this study were less likely to report potential symptoms to avoid the stigma of COVID-19, as fear of the stigma associated with COVID-19 is a problem for many people (29, 51, 52).

Worrying about COVID-19 was significantly associated with one's level of education. For example, participants with pre-university education or university education were 7.94 times and 4.14 times more worried about COVID-19 than the post-graduate group, respectively. Similarly, a study by Megatsari et al., revealed that participants with lower education levels were 3.12 times more likely to be concerned than those with a higher education level (53). The reason for this is unknown, but it is possible that the more-educated participants had greater confidence in the effectiveness of personal protection against COVID-19.

Females were 3.23 times more likely to wear a face mask during the COVID-19 pandemic than were men. This finding was supported by prior research, in which women were found to report more compliance with COVID-19 precautionary instructions than men (45, 54, 55).

## Limitations

There are several limitations of this study to acknowledge. First, given the traveling restrictions during the COVID-19 pandemic, the researcher used a cross-sectional online survey and non-random sampling. Second, the researcher collected data only from educated people who had Internet access and were willing to participate in the study; thus, this study did not target expatriate laborers and individuals who have not adopted technology and social media, which may exclude older people. Third, it was noted that all dimensions of the KAP achieved high scores. These high scores could be attributed to the desire to provide socially acceptable responses. Immigrants and older people might be expected to achieve lower KAP scores and failing to reach them may contribute to the current KAP scores. Finally, all participants came from the Jazan region. Thus, the results may not be generalizable to the rest of Saudi Arabia. Despite these limitations, the current study contributes significantly to COVID-19 research.

## Conclusion

Most of the study participants had good knowledge, positive attitudes, and effective practices toward COVID-19. However, the results imply that the COVID-19 awareness activities must be prioritized for the less educated and lower-income population. The findings of this study may help guide future awareness resources to the groups most in need in the Jazan region, particularly as the COVID-19 situation develops and changes. Further research should consider the groups omitted from this study, including immigrants and the elderly who have not adopted social media and technology.

## Data Availability Statement

The raw data supporting the conclusions of this article will be made available by the author, without undue reservation.

## Ethics Statement

The studies involving human participants were reviewed and approved by the Research Ethics Committee of Jazan University, Saudi Arabia. The patients/participants provided their written informed consent to participate in this study.

## Author Contributions

MA is the author responsible for the conception, design, acquisition of data, analysis and interpretation of data, drafting of the article, and critical revision of the article for important intellectual content and, read and approved the submitted version.

## Funding

A special thanks goes to Maram Almalki for her assistance throughout this study, including English editing.

## Conflict of Interest

The author declares that the research was conducted in the absence of any commercial or financial relationships that could be construed as a potential conflict of interest.

## Publisher's Note

All claims expressed in this article are solely those of the authors and do not necessarily represent those of their affiliated organizations, or those of the publisher, the editors and the reviewers. Any product that may be evaluated in this article, or claim that may be made by its manufacturer, is not guaranteed or endorsed by the publisher.
