# Supplementary material for: Knowledge, Attitudes, and Practices Toward COVID-19 Among the General Public in the Border Region of Jazan, Saudi Arabia: A Cross-Sectional Study
Source: Front Public Health. 2021 Dec 8;9:733125. doi: 10.3389/fpubh.2021.733125 (PMC8692766; doi:10.3389/fpubh.2021.733125)
Supplement: Supplementary file 1 [file Table_1.DOCX]

Supplementary Material

# Supplementary file1: KAP Questionnaire of COVID-19

**Do you live or work in Jazan, Saudi Arabia?**

1  Yes (please continue to the next question)

2  No (you may stop here, thank you)

**Do you agree to participate in this study?**

1  Yes (please start the questionnaire on the next section)

2  No (you may stop here, thank you)

Answering all questions will enable us to analyze the data of this questionnaire and to obtain accurate findings. Please ensure your full participation. Thank you.

**PART ONE: Background Information**

1. **Your gender?**

1  Male

2  Female

1. **Your age?** (_______________________)
2. **Your marital status?**

1  Married

2  Never-married

3  Other (Please specify__________________)

1. **Your nationality?**

1  Saudi

2  Non-Saudi (Please specify__________________)

1. **Your HIGHEST level of education?**

1  Primary

2  Intermediate

3  Secondary / High school

4  Post-secondary Diploma

5  Bachelor's degree

6  Master’s degree

7  Doctoral degree (PhD / Professional Doctorate)

8  Other (Please specify__________________)

1. **Your occupation?** (________________________)
2. **Income per month in Saudi Riyal, SR?**

1  SR 10,000 or less

2  > SR 10,000 to 20,000

3  > SR 20,000

1. **Did you attend an online/onsite health education activity related to COVID-19?**

1  Yes

2  No

1. **Which city / governorate do you live in?** (________________________)
2. **What are your sources of information about COVID-19? (Please choose the appropriate answers).**

1  Social Media

2  Public News and Television

3  Ministry of Health Website

4  World Health Organization Website

5  Internet Search Engines

6  Family

7  Ministry of Health Mobile Messages

8  Other (Please specify__________________)

**PART TWO: Knowledge, Attitudes and Practices**

**Knowledge (K): Personal knowledge regarding COVID-19.**

|  | Yes | No | Not sure |
| --- | --- | --- | --- |
| **K1: COVID-19 has spread in many countries worldwide.** |  |  |  |
| **K2: There is a licensed vaccine available for protection against COVID-19.** |  |  |  |
| **K3: All cases of COVID-19 develop to severe conditions.** |  |  |  |
| **K4: The probability of contracting COVID-19 increases in mass gathering.** |  |  |  |
| **K5: Confirmed cases or deaths from COVID-19 have occurred in Saudi Arabia.** |  |  |  |
| **K6: It is believed that the primary source of COVID-19 is zoonotic (emerged from an animal source).** |  |  |  |
| **K7: There are diagnostic tests available for COVID-19.** |  |  |  |

**K8: Where did COVID-19 appear for the first time?**

1  Bangkok in Thailand

2  Wuhan in China

3  Seoul in South Korea

4  Beijing in China

5  Rome in Italy

6  I do not know

**K9: Which of the following is the cause of COVID-19?**

1  Fungus

2  Bacteria

3  Virus

4  I do not know

**K10: What is the currently confirmed incubation period for COVID-19? (2–14 days).**

1  Less than 2 days

2  2 -5 days

3  2-14 days

4  I do not know

**K11: COVID-19 symptoms may include: (Please choose the appropriate answers).**

1  Fever

2  Dry cough

3  Shortness of breath

4  Tiredness

5  Sore throat

6  Congested or runny nose

7  Diarrhea

8  Loss of taste or smell

9  I do not know

**K12: COVID-19 may affect all age groups, but which groups are at increased risk for this disease? (Please choose the appropriate answers).**

1  Older people

2  People with pre-existing medical conditions

3  Pregnant women

4  Young people

5  Children

6  I do not know

**K13: Is there a specific medication for COVID-19? (Please choose the appropriate answers).**

1  There is no specific medication

2  Medical and nursing care with supportive medications can help most patients recover from COVID-19

3  I do not know

**K14: In more severe cases of COVID-19, infection can cause:**

1  Pneumonia

2  Acute respiratory distress syndrome

3  Death

4  I do not know

**K15: People should wear face masks: (Please choose the appropriate answers).**

1  If they suffer from respiratory symptoms (coughing or sneezing)

2  During a direct contact with someone who has respiratory symptoms

3  If they stay at home

4  I do not know

**K16: During the COVID-19 pandemic, anyone with fever, cough, or shortness of breath must: (Please choose the appropriate answers).**

1  Wear a face mask

2  Isolate himself/herself from others

3  Call the Ministry of Health hotline (937)

4  Visit the nearest health facility while taking the necessary precautions

5  Live a usual social life

6  I do not know

**K17: Which of the following are prevention measures against COVID-19? (Please choose the appropriate answers).**

1  Wash hands with water and soap for 40 seconds or rub hands with alcohol-based sanitizers for 20 seconds

2  Avoid touching the eyes, nose, and mouth with unwashed hands

3  Avoid contacts with infected people

4  Cover mouth and nose when coughing or sneezing

5  Avoid shaking hands

6  Wash hands or use hand sanitizer after touching items in public places.

7  Close windows at home

8  Wear gloves all times

9  I do not know

**Attitudes (A): Personal attitudes towards COVID-19.
Please answer according to your attitudes towards the following statements.**

|  | Strongly Agree | Agree | Neutral | Disagree | Strongly Disagree |
| --- | --- | --- | --- | --- | --- |
| **A1: I am worried about myself or a family member becoming infected.** |  |  |  |  |  |
| **A2: If a vaccine is available, I will take it.** |  |  |  |  |  |
| **A3: If I develop symptoms of COVID-19, I will inform the health authority.** |  |  |  |  |  |
| **A4: I am confident that the current protection measures taken by the government are sufficient to control the disease.** |  |  |  |  |  |

**Practices (P): Personal practice towards COVID-19
Please respond according to your actual practices.**

|  | Yes | No |
| --- | --- | --- |
| **P1: I avoid leaving the house except for necessities.** |  |  |
| **P2: I wear a face mask in crowded places.** |  |  |
| **P3: I avoid touching or shaking hands with others.** |  |  |
| **P4: I am doing my best to maintain social distancing.** |  |  |
| **P5: I do not touch my mouth, nose or eyes with unwashed hands.** |  |  |
| **P6: I wash my hands with soap and water for 40 seconds, or I rub my hands with alcohol-based sanitizers for 20 seconds.** |  |  |

**In the space below, please write any additional notes or information that you believe will enhance the results of the study.**

THANK YOU FOR COMPLETING THIS SURVEY.

# أستبانة المعرفة والمواقف والممارسات المتعلقة بكوفيد-19

**هل تعيش أو تعمل في منطقة جازان بالمملكة العربية السعودية؟**

1  نعم (من فضلك انتقل للسؤال التالي)

2  لا (بإمكانك التوقف هنا، شكرًا لك)

**هل توافق على المشاركة في هذه الدراسة؟**

1  نعم (من فضلك انتقل للسؤال التالي)

2  لا (بإمكانك التوقف هنا، شكرًا لك)

**الإجابة على كافة الأسئلة سيمكننا من التحليل الجيد لنتائج هذه الاستبانة. نأمل الإجابة على جميع الأسئلة. شكرًا لك.**

**الجزء الأول: معلومات أساسية**

**1. جنسك؟**

1  ذكر

2  أنثى

**2. عمرك؟ (____________________)**

**3. الحالة الاجتماعية**

1  متزوج

2  غير متزوج

3  أخرى **(من فضلك حدد ____________________)**

**4. جنسيتك؟**

1  سعودي

2  غير سعودي **(من فضلك حدد ____________________)**

**5. المستوى التعليمي؟**

1  ابتدائي

2  متوسط

3  ثانوي

4  دبلوم بعد الثانوي

5  بكالوريوس

6  ماجستير

7  درجة الدكتوراه

8  أخرى **(من فضلك حدد ____________________)**

**6. الوظيفة الحالية؟ (____________________)**

**7. المستوى التعليمي؟**

1  يساوي أو أقل من 10,000 ريال سعودي

2  ما بين 10,000 – 20,000 ريال سعودي

3  أعلى من 20,000 ريال سعودي

**8. هل التحقت بأي برنامج تثقيفي مباشر أو عن بعد عن وباء كورونا المستجد؟**

1  نعم

2  لا

**9. ما هي المنطقة التي تعيش فيها؟ (____________________)**

**10. ما هي المدينة أو المحافظة التي تعيش فيها؟ (____________________)**

**11. ما هي مصادر معلوماتك عن مرض كورونا المستجد ( كوفيد-19)؟ (اختر الإجابات المناسبة من القائمة التالية)**

1  تطبيقات التواصل الاجتماعي

2  الاخبار العامة والتلفزيون

3  الموقع الإلكتروني لوزارة الصحة

4  الموقع الإلكتروني لمنظمة الصحة العالمية

5  محركات البحث على الإنترنت

6  أفراد الأسرة

7  رسائل الجوال من وزارة الصحة

8  أخرى **(من فضلك حدد ____________________)**

**الجزء الثاني: المعارف والمواقف والممارسات**

**المعرفة الشخصية بشأن مرض كورونا المستجد (كوفيد-19)**

|  | نعم | لا | غير متأكد |
| --- | --- | --- | --- |
| 1. **كوفيد-19 انتشر في العديد من البلدان حول العالم** |  |  |  |
| 1. **يتوفر لقاح مرخص للوقاية من كوفيد-19** |  |  |  |
| 1. **تتحول جميع حالات كوفيد-19 إلى حالات شديدة** |  |  |  |
| 1. **احتمالية الإصابة بــ كوفيد-19 تزيد في التجمعات الكبيرة** |  |  |  |
| 1. **ظهرت حالات إصابة مؤكدة أو وفيات بسبب كوفيد-19 في المملكة العربية السعودية** |  |  |  |
| 1. **يعتقد أن المسبب الأساسي لمرض كوفيد-19 حيواني المنشأ، ولكنه الآن ينتقل من إنسان لآخر** |  |  |  |
| 1. **يوجد اختبارات لتشخيص كوفيد-19** |  |  |  |

**8. أين ظهر مرض كوفيد-19 لأول مرة؟**

1  بانكوك في تايلند

2  ووهان في الصين

3  سيول في كوريا

4  بكين في الصين

5  روما في إيطاليا

6  لا أدري

**9. أي مما يلي هو المسبب للإصابة بمرض كوفيد-19؟**

1  الفطريات

2  البكتيريا

3  الفيروسات

4  لا أدري

**10. ما هي فترة الحضانة المؤكدة حتى الآن لمرض كوفيد-19؟**

1  أقل من يومين

2  2 إلى 5 أيام

3  2 إلى 14 يوم

4  لا أدري

**11. أعراض كوفيد-19 قد تشتمل على ما يلي: (اختر الإجابات المناسبة من القائمة التالية)**

1  حمى

2  كحة جافة

3  قصور أو صعوبات في النفس

4  آلام جسدية وتعب

5  آلام الحلق

6  احتقان الأنف أو الرشح

7  إسهال

8  فقدان حاسة التذوق أو الشم

9  لا أدري

**12. كوفيد-19 قد يصيب جميع الفئات العمرية، ولكن ماهي الفئات الأكثر عرضة للإصابة بهذا المرض. (اختر الإجابات المناسبة من القائمة التالية)**

1  كبار السن

2  الأشخاص الذين يعانون من مشاكل صحية

3  النساء الحوامل

4  الشباب

5  الأطفال

6  لا أدري

**13. هل يوجد دواء مخصص لعلاج كوفيد-19؟ (اختر الإجابات المناسبة من القائمة التالية)**

1  ليس هنالك دواء مخصص

2  الرعاية الطبية والتمريضية مع الأدوية المساعدة يمكن أن تساعد معظم المرضى على التعافي من كوفيد-19

3  لا أدري

**14. في حالات كوفيد-19 الشديدة، يمكن للعدوى أن تسبب: (اختر الإجابات المناسبة من القائمة التالية)**

1  التهاب رئوي

2  متلازمة الالتهاب الرئوى الحاد

3  الوفاة

4  لا أدري

**15. يجب لبس كمامات الوجه في الحالات التالية: (اختر الإجابات المناسبة من القائمة التالية)**

1  عند الإصابة بأعراض تنفسية مثل الكحة والعطاس

2  عند المخالطة المباشرة لشخص لديه أعراض تنفسية

3  أثناء البقاء في المنزل

4  لا أدري

**16. أي شخص أثناء جائحة كوفيد-19 يعاني من حمى وكحة أو قصور في التنفس يجب أن: (اختر الإجابات المناسبة من القائمة التالية)**

1  يلبس كمام وجه

2  يعزل نفسه عن الآخرين

3  يتصل على الخط الساخن لوزارة الصحة (937)

4  يتوجه لأقرب منشأة صحية بعد أخذ الاحتياطات اللازمة

5  يمارس حياته الاجتماعية بصورة طبيعية

6  لا أدري

**17. أي مما يلي تعتبر تدابير وقائية ضد كوفيد-19؟ (اختر الإجابات المناسبة من القائمة التالية)**

1  غسل اليدين بالماء والصابون لمدة 40 ثانية أو فركهما بمطهرات الكحول لمدة 20 ثانية

2  تجنب لمس العين والأنف والفم بأيدٍ غير مغسولة

3  تجنب مخالطة الأشخاص المصابين

4  تغطية الفم والأنف عند السعال أو العطس

5  تجنب المصافحة

6  غسل اليدين أو فركهما بمطهرات الكحول بعد لمس الأشياء في الأماكن العامة

7  قفل نوافذ المنزل

8  لبس القفازات بشكل مستمر

9  لا أدري

**المواقف الشخصية من مرض فيروس كورونا المستجد (كوفيد-19)
فضلًا أجب بحسب انطباعك أو موقفك أنت من الجمل التالية.**

|  | موافق وبشدة | موافق | محايد | غير موافق | غير موافق وبشدة |
| --- | --- | --- | --- | --- | --- |
| 1. **أشعر بالقلق من احتمال إصابتي أو إصابة أفراد عائلتي بالمرض** |  |  |  |  |  |
| 1. **سأتلقى اللقاح في حال توفره** |  |  |  |  |  |
| 1. **إذا ظهرت لدي أعراض كوفيد-19، فسوف أبلغ السلطات الصحية.** |  |  |  |  |  |
| 1. **تدابير الحماية الحالية التي اتخذتها الحكومة كافية للسيطرة على المرض** |  |  |  |  |  |

**الممارسات الشخصية فيما يتعلق بمرض كورونا المستجد (كوفيد-19)
فضلًا أجب بحسب ممارساتك الفعلية.**

|  | نعم | لا |
| --- | --- | --- |
| 1. **لا أغادر المنزل إلا للضرورة** |  |  |
| 1. **أرتدي كمام الوجه في الأماكن المزدحمة** |  |  |
| 1. **أتجنب لمس أو مصافحة أيدي الآخرين** |  |  |
| 1. **أبذل قصارى جهدي للحفاظ على التباعد الاجتماعي** |  |  |
| 1. **لا ألمس فمي أو أنفي أو عيني بأيديٍ غير مغسولة** |  |  |
| 1. **أغسل يديّ بالصابون والماء لمدة 40 ثانية، أو أفركهما بالمطهرات الكحولية لمدة 20 ثانية** |  |  |

**في المساحة أدناه، فضلاً أكتب أي ملاحظات أو معلومات إضافية تعتقد أنها ستعزز من نتائج الدراسة.**

شكرًا لك للمشاركة في هذه الاستبانة
